# MSOME/IMSI versus epigenetic marks: doubts in the past for hopes in the future

**DOI:** 10.5935/1518-0557.20220054

**Published:** 2022

**Authors:** Jose Gonçalves Franco Jr, Joao Batista A Oliveira

**Affiliations:** 1 Center for Human Reproduction Prof. Franco Junior, Ribeirão Preto/SP - Brazil; 2 Paulista Center for Diagnosis Research and Training, Ribeirao Preto/SP - Brazil; 3 Editorial Consultant - JBRA Assisted Reproduction

**Keywords:** MSOME, IMSI, epigenetic marks, sperm vacuoles

Since 2008, our group has published studies showing a relationship between spermatozoa with vacuoles and increased DNA fragmentation (Franco Jr *et al*., 2008; Oliveira *et al*., 2010); as well as an association between sperm with vacuoles and abnormal chromatin packing (Franco Jr *et al*., 2012). Based on these studies and those of other authors (Garolla *et al*., 2008), we decided to consider spermatozoa with vacuoles (especially those occupying ≥ 50% nuclear area) as a risk cell for transporting DNA damage, and we started to use selection at high magnification (MSOME/IMSI) as a routine for ICSI cases. During these almost 15 years we have been heavily criticized for this approach based only on basic studies, especially considering the divergence in the literature regarding its efficiency versus ICSI in improving parameters traditionally used in the evaluation of any methodology in assisted reproduction (fertilization rates, clinical pregnancy, live births, etc.). In other words, we would be intensively cultivating one more of these countless adds-on.

In 2021, Dieamant et al. published a systematic review and meta-analysis based on the birth of 3907 children born after IMSI (n=1280) and through ICSI (n=2627). The results demonstrated that IMSI decreased the incidence of structural defects compared to ICSI (*p*=0.04). However, there was no significant difference in chromosomal abnormalities (Trisomy 13;18; 21 and triple X) between children conceived after IMSI or ICSI. In principle, these data confirm that MSOME/IMSI should not be abandoned, because the identification of birth defects is not something that can be neglected. In principle, assisted reproduction methodologies should not be evaluated only by traditional outcomes, but also by how they would influence the incidence of congenital malformations. This is perhaps the minimum, but in the future epigenetic manifestations could not be ruled out. Could preventing diseases such as obesity, arterial hypertension, diabetes guide scientific thinking in choosing an assisted reproduction methodology? It would be coherent, however with regards to MSOME/IMSI there was a missing link in this scientific thinking. Bendayan *et al*. (2022) demonstrated that sperm with vacuoles carry altered epigenetic marks (H3K4me3, H3K27me3) compared to sperm without vacuoles. Finally, something that unites all these facts and awakens the need for a change in the judgment of an assisted reproduction methodology. Epigenetic prevention could be a new outcome.

Following this line of thought, MSOME/IMSI could be the first tool in assisted reproduction capable of reducing the risk of transmission of epigenetic problems through spermatozoa. Hallelujah, 15 years of employing MSOME/IMSI may have been worth it!
